# Novel variants of human herpesvirus 2 from Brazilian HIV-1 coinfected subjects

**DOI:** 10.1590/0074-02760180328

**Published:** 2018-12-03

**Authors:** Lyana Rodrigues Pinto Lima, Nathália Alves de Araújo, Alexandro Guterres, José Henrique Pilotto, Christian Niel, Vanessa Salete de Paula

**Affiliations:** 1 Fundação Oswaldo Cruz-Fiocruz, Instituto Oswaldo Cruz, Laboratório de Virologia Molecular, Rio de Janeiro, RJ, Brasil; 2 Fundação Oswaldo Cruz-Fiocruz, Instituto Oswaldo Cruz, Laboratório de Hantaviroses e Rickettsioses, Rio de Janeiro, RJ, Brasil; 3 Fundação Oswaldo Cruz-Fiocruz, Instituto Oswaldo Cruz, Laboratório de AIDS e Imunologia Molecular, Rio de Janeiro, RJ, Brasil; 4 Hospital Geral, Nova Iguaçu, RJ, Brasil

**Keywords:** Brazil, clade, co-infection, glycoprotein B, HSV-2, HIV

## Abstract

BACKGROUND Human herpesvirus 2 (HHV-2) have DNA genome with a limited genetic variability and have been classified into two clades. OBJECTIVES To identify and characterise six HHV-2 isolates derived from Brazilian women. METHODS HHV-2 isolates were performed polymerase chain reaction (PCR) and sequencing of 2250 pb of the glycoprotein B (gB) coding regions. FINDINGS Four HHV-2 isolates were classified into clade B, while the remaining two, derived from HIV-1 co-infected women, showed a notable genetic divergence (> 1%). MAIN CONCLUSION The results reveal novel HHV-2 variants. The impact of these novel variants on HHV-2 pathogenesis and HIV/HHV-2 coinfection need to be investigated.

Human herpesvirus 2 (HHV-2) is the main cause of genital herpes. The virus is mainly transmitted through sexual contact and typically causes an asymptomatic infection. Worldwide, an estimated 267 million women are living with HHV-2 infection.^1^ In Brazil, the HHV-2 is the cause of 55.3% of genital ulcer among patients from Brazilian Amazon.^2^ Previous studies showed HHV-2 prevalence rates ranged of 6.6% to 15.6% among women,^3^ ^,^ ^4^ ^,^ ^5^ 19.2 to 59.7% in women infected with HIV.^4^ ^,^ ^6^

HHV-2 is an enveloped virus belonging to the *Herpesviridae* family. The envelope is a lipid bilayer with 12 glycoproteins which are necessary for early interactions between virus and target cells.^7^ Little is known about the genetic diversity of the HHV-2 isolates circulating worldwide. The glycoprotein B (gB) coding sequence is one of the most conserved genes within the family *Herpesviridae*.^8^ Phylogenetic analyses performed with genes coding for glycoproteins G, I, E and B led to the classification of HHV-2 isolates into two clades, A and B.^8^ ^,^ ^9^ Moreover, a genetically divergent variant, called HSV-2v, has been recently characterised.^10^ ^,^ ^11^ Isolates from Europe,^8^ ^,^ ^9^ America,^10^ ^,^ ^12^ Asia^13^ and Africa^8^ ^,^ ^11^ ^,^ ^12^ ^,^ ^13^ have been characterised. However, no data are available for HHV-2 isolates circulating in Brazil.

Blood samples and/or cutaneous lesions were collected at the time of appearance of the genital lesions. This samples are from six Brazilian women (two whites, three browns and one black), aged 26 to 58 years and living in Rio de Janeiro, Brazil. Two of them were pregnant, coinfected with HIV-1 (CD4/CD8 ratios of 0.61 and 1.18, respectively), and receiving antiviral therapy.

Antibodies against HHV-2 were detected by enzyme immunoassay (Bioelisa HSV-2 IgG, Biokit, Barcelona Spain). Viral DNA was detected by real time polymerase chain reaction (PCR), as described previously.^14^ In addition, ten overlapping genome segments, covering almost all (2,250 bp) the gB encoding region (UL-27) were amplified in single round PCR assays. Each reaction was performed in a 25-μL volume containing 5 μL of DNA, 0.3 μM of each primer and one unit of GoTaq DNA polymerase (Promega, Madison, WI) under the following conditions: 95ºC for 5 min, 40 cycles of 95ºC for 30 s, 55-59ºC for 45 s, 72ºC for 90 s, followed for 10 min at 72ºC. PCR products were purified and directly sequenced with the ABI Big Dye Terminator cycle sequencing kit, version 1.1 (Applied Biosystems, Foster City, CA) and the primers used for PCR (Table I).

Multiple sequence alignments were done with sequences from this study and all the 140 HHV-2 gB sequences available in GenBank in September 2016, using MUSCLE, in the Mega 6.1 program. The best-fit evolutionary model was determined using the jModelTest version 2.0. Of the 140 HHV-2 sequences, were selected 37 HHV-2 isolates representing different countries to perform Phylogenetic relationships. The number reduction of the sequences was needed to decrease the time phylogenetic analysis performed in computer programs. Phylogenetic relationships among 37 HHV-2 isolates were estimated by the Bayesian Markov Chain Monte Carlo (MCMC) method, implemented in MrBayes, version 3.2.3. The Bayesian analysis consisted of two simultaneous independent runs of 5 million MCMC generations (burn-in of 25%). We used Tracer v.1.6 to check for convergence and adequate mixing (i.e., an estimated sample size > 200 for all relevant parameters). In order to analyse possible recombination events, the sequence alignment was analysed with Bootscan, implemented in Simplot and RPD4.^15^ ^,^ ^16^ The sequences for Bootscan analysis were grouped according to clustering of the nominal taxa seen in the phylogenetic tree for each sequence.

Six HHV-2 infected women were enrolled in this study which was approved by the Ethics Committee of the Oswaldo Cruz Institute (number: 895159/CAEE:28183314.7.0000.5248). All six participants signed an informed consent form.

All six samples tested positive for HHV-2 IgG antibodies and real time PCR. Viral load varied from 3.5 x 10^2^ to 1.5 x 10^6^ copies/mL. In this study 82% (2,250 bp) of the gB coding sequences of all six HHV-2 isolates were determined (GenBank accession numbers: KY007702, KY007703, KY007704, KY007705, KY007706 and KY007707). Phylogenetic analysis showed that the four HHV-2 Brazilian isolates (BR014, BR020, BR022 and BR041) derived from the HIV negative women showed a close genetic relationship between them (99.4-99.6% of sequence identity) and clustered with European, North American and Asian strains within clade B (Figure). Meanwhile, the two isolates (BR021 and BR123) derived from the HIV positive women were almost identical (99.8% of sequence identity) but clustered separately from isolates previously classified into clades A and B as well as from the variants HSV-2v (genetic divergence of 1.1-1.3%) with a posterior probability of 1. We suggest to provisionally classify the new class of variants represented by isolates BR021 and BR123 as HHV-2vBR.

Both isolates BR021 and BR123 showed 16 unique nucleotide substitutions when compared with all 140 HHV-2 gB sequences available in GenBank (Table II). Twelve of these mutations were G→C and C→G transversions. Fourteen were silent while the remaining two (G1411C and G1747A) resulted in Ala→Pro and Val→Ile changes, respectively. Interestingly, four of these 16 mutations were also present in the chimpanzee alphaherpesvirus (ChHV) prototype strain 105640 (Table II).

HHV-2 genome, as well as those of the other herpesviruses, is characterised by a low rate of mutation during the replication process.^17^ As gB coding region is highly conserved, a low divergence (0.2-0.5%) has been observed among the gB sequences of HHV-2 isolates worldwide.^8^ ^,^ ^13^ ^,^ ^18^ In this study, however, the divergence rates found among the gB sequences of Brazilian samples BR021 and BR123 were greater than 1%.

A new variant, showing a high degree of genetic divergence with respect to the HHV-2 reference strain in the UL30 encoding the DNA polymerase, has been recently described.^10^ This variant, called HSV-2v to distinguish it from the ‘classical’ HSV-2c isolates, was first found to circulate in Paris, France, between 2008 and 2012, and further significantly associated with an African origin and HIV co-infection.^11^ According to specific clade definition criteria, HSV-2v could be referred to as a new African HHV-2 clade. Interestingly, Brazilian HHV-2 variants BR021 and BR123 characterised in this study were derived from HIV infected persons whose ancestors were Africans (one was black, and the other was brown), suggestion the association of HHV-2 novels variants with HIV and African origin. In contrast with our results, Abrao et al.^19^ showed no specific differences regarding replication capacity and gene sequence were found when comparing HHV-2 strains from HIV-infected patients and HIV-negative patients, suggesting that HHV-2 infection are not influenced by HIV-1 infection.^19^

**TABLE I:** Oligonucleotide primers used in this study

Primer	Genome position^*^	Sequence (5´ → 3´)	Reference
gB1 For		CCCATCCCCTCGAAGAAC	Abrão et al.^(19)^
gB1 Rev	577	CAGACCCCCTTGGCGTTAAT	This study
gB2 For	424	TTCAAGGAGAACATCGCCCC	This study
gB2 Rev	744	GGGGTTGTACTTGAGGTCGG	This study
gB3 For	557	ATTAACGCCAAGGGGGTCTG	This study
gB3 Rev	885	GTAGCCGTAAAACGGGGACA	This study
gB4 For	725	CCGACCTCAAGTACAACCCC	This study
gB4 Rev	1164	GGTGAAGGTGGTCGAGATGG	This study
gB5 For	1008	GCTGACGACCCCAAGTTTA	This study
gB5 Rev	1487	TCGATCGAGGAGGTGGTCTT	This study
gB6 For	1158	CCTTCACCACCAACCTGACC	This study
gB6 Rev	1532	CGTGGCGCTGTATGTGGTTA	This study
gB7 For	1464	GCATCAAGACCACCTCCTCG	This study
gB7 Rev	1912	GAAGATGAAGTAGCGCCGGT	This study
gB8 For	1604	CGAGCTGACTCTCTCGGAACG	This study
gB8 Rev	2035	CACAAACTCGTGGTCCTCCA	This study
gB9 For	1916	GGGGCTACGTGTACTTCGAG	This study
gB9 Rev	2410	ACAGGGCCTTCATGGGATTG	This study
gB10 For	2391	CAATCCCATGAAGGCCCTGT	This study
gB10 Rev	2606	GTTGGTGACCTTGGAGCTGA	This study

*: numbering from the initiation codon of the glycoprotein B (gB) coding region.

**Phylogenetic relationships:**
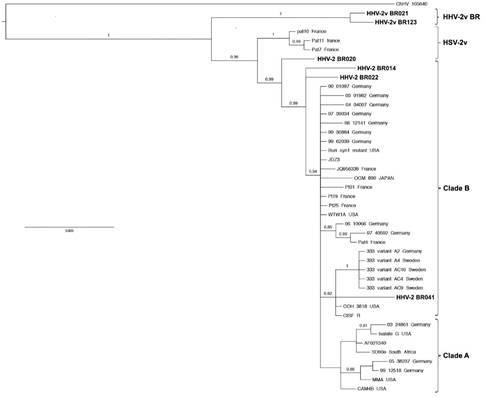
among human herpesvirus 2 (HHV-2) based on Bayesian analysis of genetic distances generated from comparisons of a 2,250-bp fragment of the glycoprotein B (gB) sequences. The scale bar indicates an evolutionary distance of 0.003 substitutions per position. Numerical values (≥ 0.7) at the nodes indicate posterior probability replicates that supported by the interior branch. The Tamura 3-parameter model with gamma-distributed rate heterogeneity (T92 + G) was selected as the best-fit evolutionary model. Clades A and B are in accordance with previous studies.^8^ ^,^ ^9^

**TABLE II:** Specific mutations found in the glycoprotein B (gB) coding regions of two human herpesvirus 2 (HHV-2) isolates derived from Brazilian HIV co-infected women

Nucleotide change^*a*^	Presence in ChHV prototype^*b*^	Amino acid change
G1254C	No	-
C1323G	No	-
G1411C	No	A471P
C1617G	No	-
C1668G	No	-
C1683G	No	-
G1731C	No	-
G1734A	Yes	-
G1747A	No	V583I
G1761C	Yes	-
G1767C	No	-
C1816T	Yes	-
C1836G	No	-
C1857G	No	-
G1972A	Yes	-
G2001C	No	-

*a*: numbering from the initiation codon of the gB coding region; *b*: number access of chimpanzee alphaherpesvirus (ChHV) prototype: NC_023677.1.

Preliminary results have suggested that HSV-2v may have acquired genomic segments from chimpanzee alphaherpesvirus (ChHV) by recombination.^11^ Here we demonstrated the existence of two variants (tentatively called HHV-2vBR) showing four nucleotide substitutions absent in all HHV-2 isolates but present in the simian virus ChHV (Table II). Both Brazilian (HHV-2vBR) variants are more closely related to ChHV (and more distant to HSV-2c) than are the HSV-2v isolates pat10, pat11 and pat7 (Figure). Whether BR021 or BR123, or both, resulted from recombinant events between human and simian herpesviruses remains to be determined. Ruling out the recombination hypotheses, the topology of the phylogenetic tree (Figure) suggests that the divergence between the Brazilian variants and the other isolates, including HSV-2c and HSV-2v, may have occur at remote times. Recombination between nucleotide sequences is a major process influencing the evolution of most viruses, but no sign of recombination was found in our dataset.

In conclusion, this study reveals the occurrence, in Brazil, of two types of HHV-2 isolates. Beside ‘classical’ isolates, derived from patients non coinfected with HIV, two variants (HHV-2vBR) showing some similarity with previously described HSV-2v variants were found to circulate. As their similar, HHV-2vBR variants were identified in patients coinfected with HIV of African ancestry. In this study, nucleotide sequencing of more than one genome region was not possible, due to the low amounts of virus (variants HHV-2vBR were derived from serum, not lesion samples). More epidemiological and molecular studies are required to confirm the existence of a new clade or genogroup and to investigate the impact of such novel variants in the pathogenesis of HHV-2 infection and HIV/HHV-2 co-infection.
